# Managing the cut end of a K wire

**DOI:** 10.4103/0970-0358.63943

**Published:** 2010

**Authors:** G. Vishwanath

**Affiliations:** INHS Asvini, Colaba, Mumbai, India

Sir,

Kirschner wires are commonly used in plastic surgery. What to do with the protruding cut end of a Kirschner wire is the question. Left unattended, the sharp cut end can cause trauma (even ocular injury) and can tear clothing.

1. It can be cut very short and driven in further, thus burying the cut end under the surface. Removal, however, will require a small procedure.
2. Commonly, the cut end is retroflexed fully so that the sharp cut end does not point outwards. However, the cut end often catches on clothing (particularly when the hand is being withdrawn from the pocket).
3. It can be covered by a cork or stopper. Babu[1] has used the rubber cap on the plunger of a plastic syringe for the purpose.
4. A short length of tube can be used for the purpose. Hu[2] has used a Nelaton's catheter for this purpose. A patented sleeve device (US Patent 4976712) exists that prevents the migration of the pin while rendering the cut end harmless.
5. Commercially available devices to attach to the cut end of the K wire include the Jurgan's ball device (Jurgan Development and Mfg.), the K-Fix pin protector device (Integra) and the ChiroKlip (Synerception).

**Figure 1:**
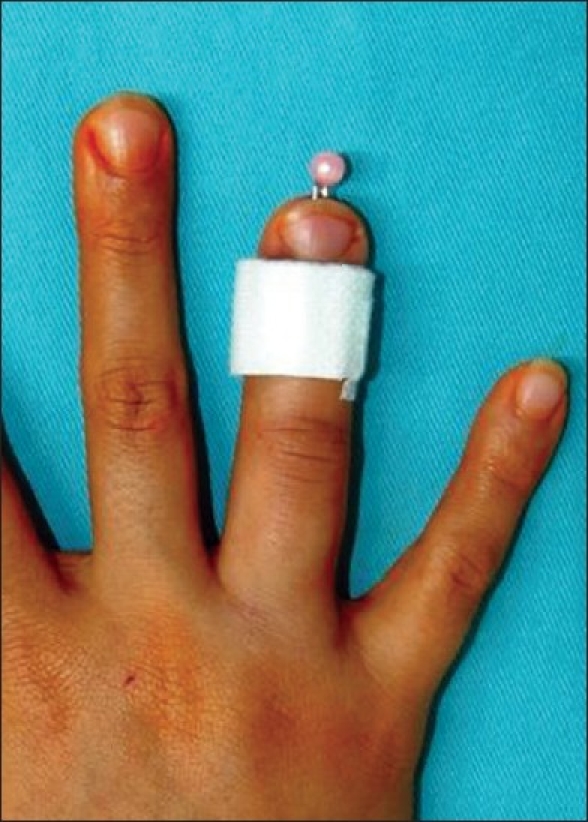
A bead on the end of a K wire

A simple and effective way to deal with the problem is as follows:

The protruding end can be cut to 5 mm or so and a plastic bead can be threaded on it and fixed with a drop of superglue (Fevikwik) rendering it safe [Figure 1]. It does not catch on clothing. The method is routinely used on all K wires at our centre and is very successful. A variety of beads are used depending on the thickness of K wire used, including those wires used in conjunction with external fixators.

An added advantage is that removal of the K wire is easy. The bead is grasped by hand and twisted and the wire is removed easily. No instrument is required. The bead remains firmly adherent to the pin during removal.
